# Exploring the needs and experiences of contact tracing staff during the COVID-19 pandemic in Ireland

**DOI:** 10.1371/journal.pone.0298799

**Published:** 2024-03-08

**Authors:** Hugh Fulham-McQuillan, Róisín O’Donovan, Claire M. Buckley, Philip Crowley, Brynne Gilmore, Jennifer Martin, Eilish McAuliffe, Gregory Martin, Gemma Moore, Mary Morrissey, Éidín Ní Shé, Mary Clare O’Hara, Mary Rose Sweeney, Patrick Wall, Aoife De Brún

**Affiliations:** 1 UCD Centre for Interdisciplinary Research, Education, and Innovation in Health Systems (UCD IRIS), School of Nursing, Midwifery & Health Systems, University College Dublin, Dublin, Ireland; 2 Centre for Positive Psychology and Health, Royal College of Surgeons in Ireland (RCSI), Dublin 2, Ireland; 3 School of Public Health, University College Cork, Cork, Ireland; 4 Team Strategy and Research Directorate, Health Service Executive, Dublin, Ireland; 5 National Quality and Patient Safety Directorate, Health Service Executive, Dublin, Ireland; 6 Health Protection Surveillance Centre, Health Service Executive, Dublin, Ireland; 7 National Health Intelligence Unit, Research & Evidence, Health Service Executive, Dublin, Ireland; 8 Graduate School of Healthcare Management, Royal College of Surgeons in Ireland (RCSI), Dublin 2, Ireland; 9 Research and Development, Strategy and Research, Health Service Executive, Dublin, Ireland; 10 Faculty of Nursing & Midwifery, Royal College of Surgeons in Ireland (RCSI), Dublin 2, Ireland; 11 School of Public Health, Physiotherapy and Sports Science, University College Dublin, Dublin, Ireland; Stamford Health, UNITED STATES

## Abstract

**Background:**

Contact tracing is a key component in controlling the spread of COVID-19, however little research has focused on learning from the experiences of contact tracing staff. Harnessing learning from those in this role can provide valuable insights into the process of contact tracing and how best to support staff in this crucial role.

**Methods:**

Thematic analysis was used to analyse 47 semi-structured interviews conducted with contact tracing staff via telephone or Zoom at three time points in 2021: March, May and September-October.

**Results:**

Six themes related to the contact tracing role were identified, including training, workforce culture, systems issues, motivation and support. While initially nervous in the role, participants were motivated to contribute to the pandemic response and believed the role provided them with valuable transferable skills. Participants described the training as having improved over time while desiring more proactive training. Sources of frustration included a perceived lack of opportunity for feedback and involvement in process changes, feelings of low autonomy, and a perception of high staff turnover. Participants expressed a need for improved communication of formal emotional supports. Increased managerial support and provision of opportunities for career advancement may contribute to increased motivation among staff.

**Conclusions:**

These findings identify the experiences of contact tracing staff working during the COVID-19 pandemic, and have important implications for the improvement of the contact tracing system. Recommendations based on learning from participants offer suggestions as to how best to support the needs of contact tracing staff during a pandemic response.

## Introduction

The World Health Organization (WHO) describes contact tracing as a key component in the approach to manage and contain the COVID-19 pandemic [1]. In recognition of this, the Irish health system rapidly developed and implemented a national COVID-19 contact tracing system [2]. A recent systematic review found that along with widespread testing and quarantine, contact tracing has been successful in reducing the incidence of COVID-19 and COVID-19 related deaths [3]. Poor contact tracing has been implicated in prolonging the duration of previous infectious disease outbreaks [4]. In the dynamic situation of the COVID-19 pandemic, optimising the process of contact tracing is necessary in order to reduce the delay in testing individuals for COVID-19 and controlling the spread of the virus both during the initial stages of an epidemic as well as during de-escalation of physical distancing [5,6]. As such, it is important to continuously learn and adapt processes through research in order to inform good practices, and to meet the needs of contact tracing staff and the public.

The contact tracing role requires a number of skills and practices including clear communication, active listening, negotiation and decision making [4], the ability to impart easily understood public health advice in line with the current guidelines, in addition to the accurate collection and management of data [7]. Contact tracing can be an emotive experience for members of the public who have developed COVID-19 or have been exposed to it [8]. Contact tracers need to build trust and offer support to the individuals they are calling [9]. In a recent qualitative study, contact tracers for COVID-19 underscored the importance of engaging authentically with clients during calls by showing empathy and building trust and rapport with the people they were calling [10]. This is particularly necessary as COVID-19-exposed individuals may also be experiencing other issues caused by the pandemic, such as anxiety, depression, grief, anger, intimate partner violence, health problems, food insecurity, and/or unemployment [11].

The development of the COVID-19 contact tracing system in Ireland was initially achieved through the redeployment of public service employees (largely healthcare and defence force) and volunteer contact tracers (many of whom were university staff), across eight contact tracing centres (CTCs) nationally, five of which were based in universities [12]. The volume of tracing required, coupled with an anticipated reduction in the availability of university staff (during the academic semester) prompted a recruitment drive. From June to August 2020, 300 dedicated staff were recruited with plans to recruit a further 500 [13]. By the end of September, 2021, recruitment had been extended to those from a variety of backgrounds, including retirees, and those with and without healthcare/clinical training and these newly recruited contact tracers were formally employed as staff [14].

In line with international examples [15], contact tracers receive short induction training [2], which took place over one day in the first three weeks of the contact tracing operation, and which expanded into three days thereafter and are often supported by more experienced tracers, but some may lack previous experience of health care and/or dealing with sensitive issues. This has significant implications for the health and well-being of contact tracers themselves. While the contact tracer training in Ireland currently offers self-care advice and national and local level supports including a psychological first aid service, research to understand the real-time impact of this work on staff is necessary to understand better how we can most appropriately support individuals in this crucial role.

There is a lack of international research on learning from the experiences of contact tracers. Examining the experiences of contract tracers and harnessing learning from those engaged in this role can provide valuable insights. These insights could identify emerging needs and issues related to the contact tracing process and adapt to respond to the shifting needs of staff and the public in this dynamic context [7,16,17]. The research question guiding this study therefore was: what are the experiences, challenges and needs identified by contact tracers working during the COVID-19 pandemic?

## Materials and methods

### Study context

**Fig 1:**
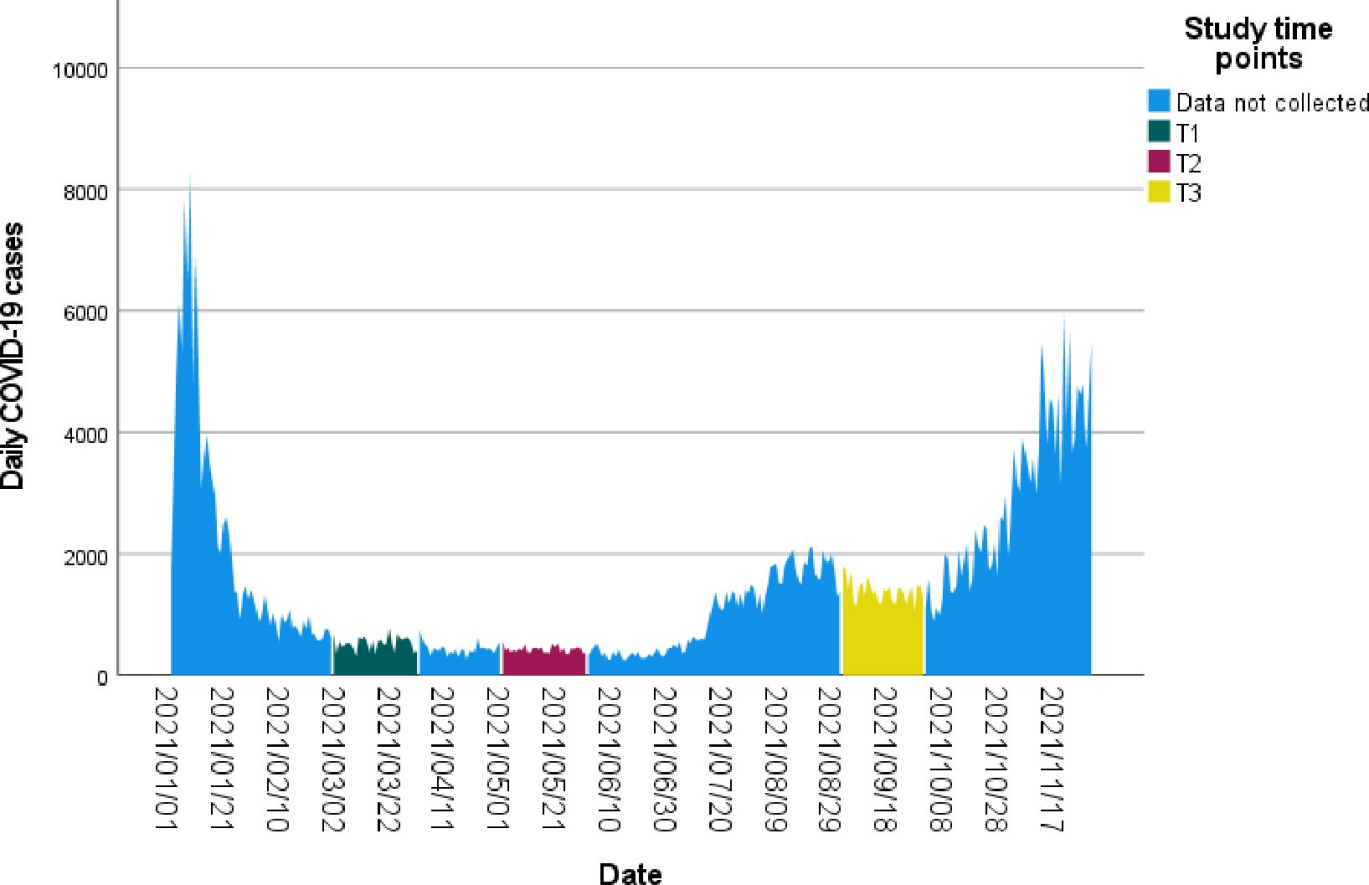
Study timeline.

The first case of COVID-19 in Ireland was diagnosed in February 2020 [18]. Ireland subsequently experienced its first significant wave of COVID-19 cases in March 2020, the second in August 2020 and the third in November 2020 [19]. As can be seen in Fig 1., data for the first time point of this study (T1) were collected after the third wave subsided in March 2021. Data were again collected in May 2021 (T2), and in September-October 2021 (T3), a month before the beginning of the fourth wave. A ransomware attack on the Health System Executive’s IT system in May 2021 [20] impacted data collection resulting in a reduced number of participants at T2 and the need to prolong recruitment in T3 into October 2021. This attack resulted in healthcare professionals losing access to all Health Service Executive (HSE) provided IT systems for approximately six weeks though several areas did not have fully operational IT access for several months.

**Fig 2:**
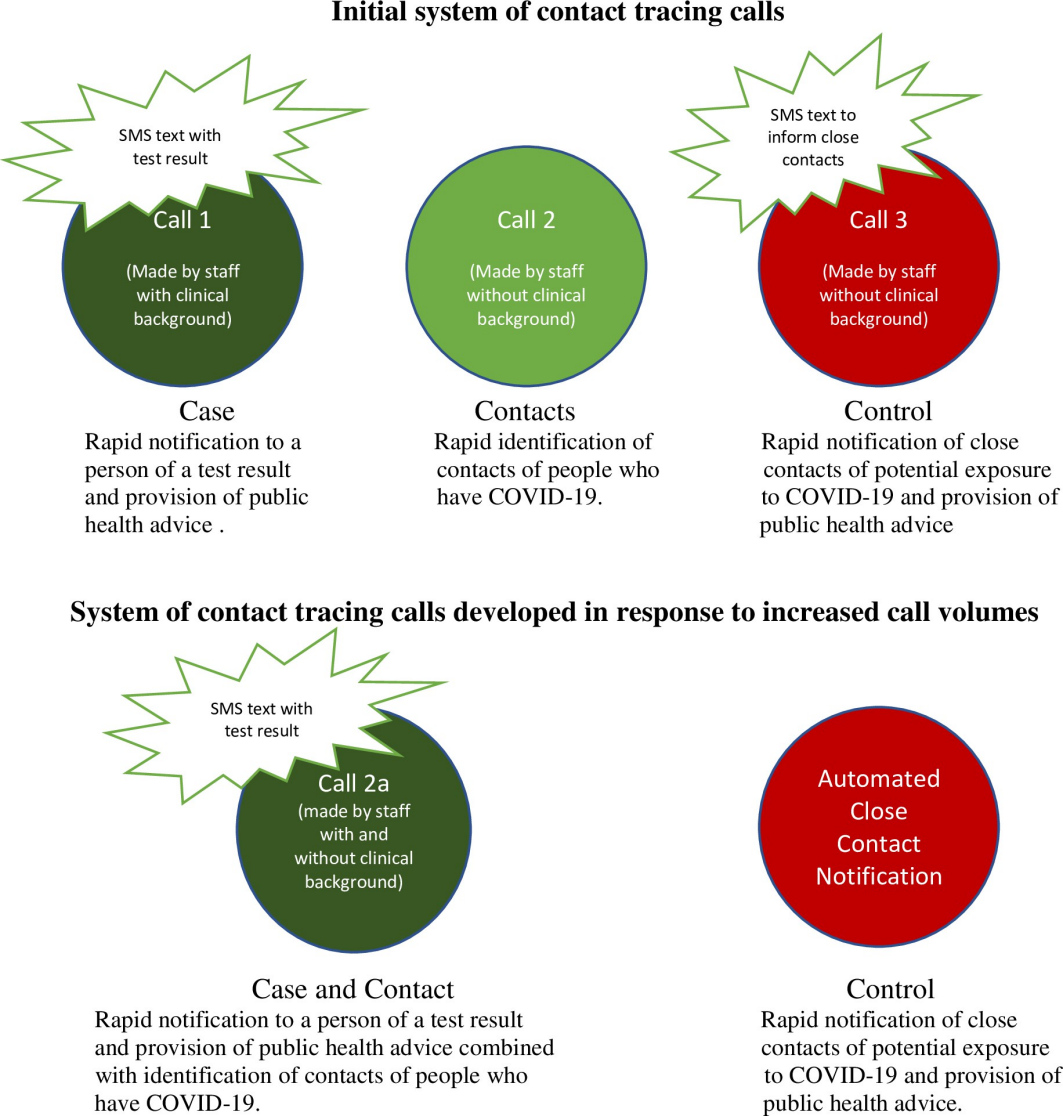
Evolution of contact tracing communications Adapted from [23].

Training for contact tracing staff followed a quality improvement approach [2], which included continual evaluation of the training programme and delivery, and adaptations reflecting user feedback. Online training platforms including an open-source learning platform, a dedicated online development and learning platform, and email communication were used to deliver ongoing information updates. While the workload of contact tracers was distributed based on prior experience and need at the time, and this allocation of work was continuously reviewed, there was a broad delineation of duties. As seen in Fig 2. those who had clinical backgrounds were recruited as contact tracers who contacted and provided public health advice to those who tested positive for COVID-19 (Call 1). Those without clinical backgrounds were recruited as contact tracers who made a second call to those who tested positive to collect the names of their contacts (Call 2) and also made calls to close contacts of those who had tested positive to inform them of their status as close contacts, provide them with public health advice and to refer them for testing (Call 3) [21]; a further function of these calls was to gather data on symptoms and possible sources of infection for use by public health staff.

A minority of contact tracers worked remotely. By the time this study commenced, all contact tracing staff were paid employees. Salaries varied depending on a number of factors including previous experience and background as a clinical and healthcare professional, with contact tracers who had clinical backgrounds receiving higher salaries on average than those without clinical backgrounds [22]. In response to the surges in COVID-19 cases, the system became overwhelmed leading to delays in contacting cases and contacts and therefore more efficient ways of working were explored. After consultation with a panel of public and patient representatives, who agreed that it was appropriate to do so, text notifications were introduced so that individuals received a text notifying them that they tested positive for COVID-19 as soon as the result was sent to the Contact Tracing Management Programme (CMP) (Fig 2). This meant that people were usually notified in advance of a call 1. By the time people were called they had already received the text message so were aware of their diagnosis and had a chance to gather details on their close contacts. Another efficiency introduced during surge was to combine the call 1 and call 2 call types. This combined call type was named call 2a, whereby a subset of epidemiological information normally taken in call 1 was collected and all contacts identified. This combined call was made by both categories of contact tracer [2].

In relation to advising contacts and arranging tests, efficiencies introduced were to SMS the contact immediately on collection of their details at call 2, and later to provide an automated option for all contacts, whereby contacts received an SMS inviting them to use a portal to receive public health advice and to request a COVID PCR test (Fig 2). Contacts who did not engage in this process would be queued for a call 3 [2].

### Research setting and participants

This paper presents the qualitative component of a larger mixed methods study using quantitative and qualitative research methods [24]. The aim of the quantitative component of the study was to understand the psychological impact of contact tracing work on staff during the COVID-19 pandemic response, and whether this impact varied according to the demographic profile of contact tracers, and to harness this learning for improvement. The current qualitative study is a separate component using separate data and analysis which aims to better understand experiences, challenges and needs identified by contact tracing staff.

**Fig 3:**
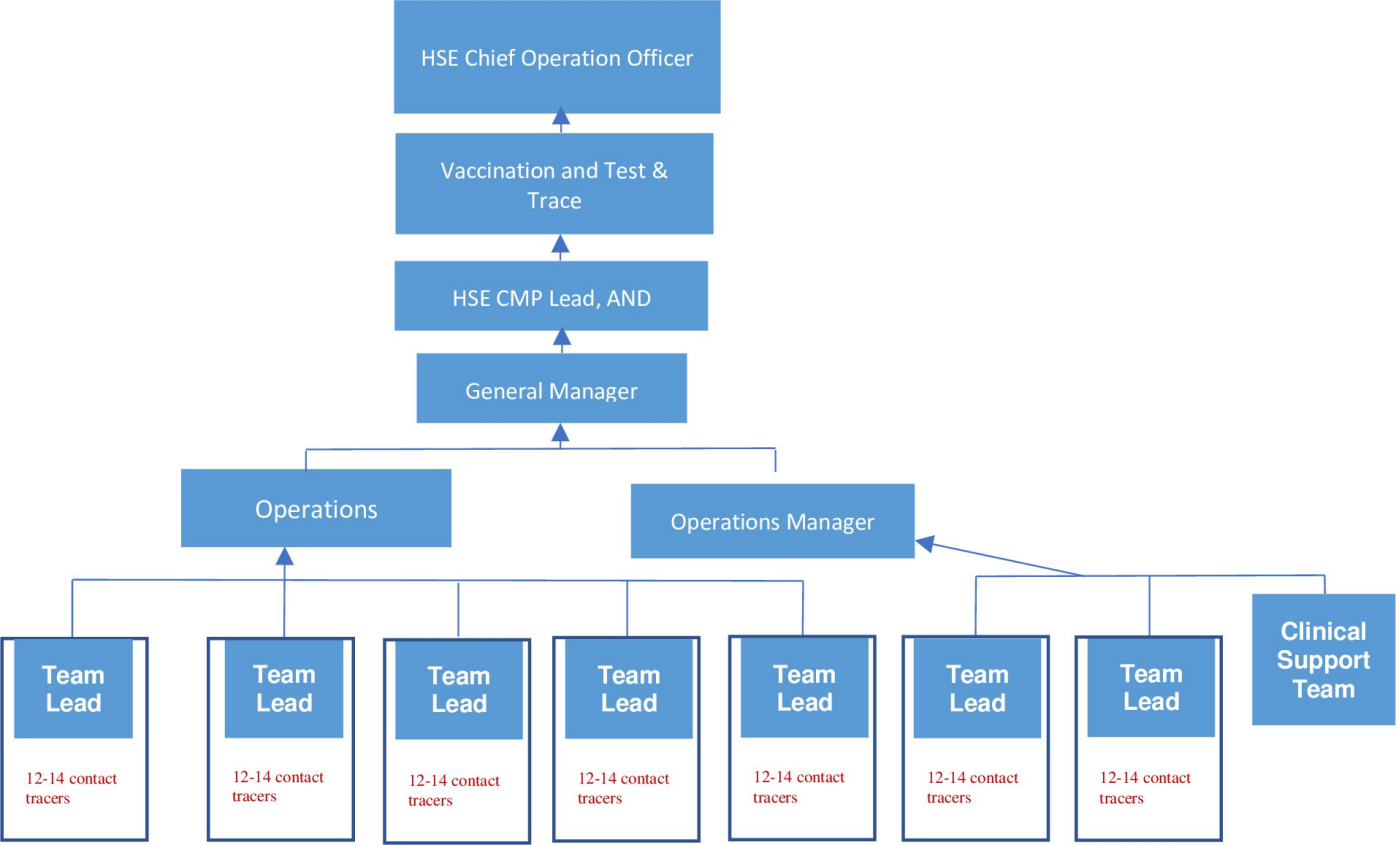
Organogram of contact tracing programme reporting lines.

A purposive sampling strategy was employed to capture a range of perspectives and experiences across contact tracers with a clinical background and those with a non-clinical background, different age groups and different length of tenure in the role of contact tracing. Participants who participated in the quantitative component of the larger study were invited to opt-in to register interest in participating in an interview about their experiences. Out of approximately 788 contact tracing staff working in Ireland during the recruitment period, a total of 47 participants took part in the present study. These participants were working as either contact tracing staff or team leads within one of six Contact Tracing Centres (CTCs) in Ireland. Team leads provide support and guidance to contact tracing staff, and report to operations managers who report a general manager and the CMP lead (Fig 3) While contact tracing staff were invited to interview at each time point, all participants were interviewed once, apart from one participant who was interviewed at both time point 1 (T1) and time point 2 (T2).

### Data collection

Individual semi-structured interviews were conducted with each participant. This method of data collection was chosen in order to gather more in-depth insights about contact tracers’ experiences than those captured in the quantitative component of the larger study, which focused primarily on the psychological impact of the role. At T1 and T2, data collection was conducted by one researcher (ROD), and by a second researcher (HFMcQ) at T3. Interviews were conducted remotely, either via Zoom or over the phone. A benefit of conducting interviews remotely was the elimination of potential geographic barriers to recruitment and potentially greater recruitment uptake due to participants not having to factor in travel time to participate. The interview topic guide was developed in collaboration with members of the Irish national health service COVID-19 Contact Management Programme and includes open-ended questions regarding participants’ experiences in their role, including experiences of training, ongoing support, and reflections of their time in their role.

### Data analysis

#### Thematic analysis

Thematic analysis, as described by Braun and Clarke [25] was employed. This involved “identifying, analysing, and reporting patterns (themes) within data” [25]. Analysis was supported by the data management programme NVivo 12 [QSR International, 2015]. Initial data analysis began after the first interview was performed throughout the data collection process at each time point. Interviews were transcribed verbatim and a brief description and summary was written, including notes on key themes, any potential amendments needed in future interviews, and the researcher’s reflections following the interview.

Each interview was then coded per process outlined by Braun and Clarke [25]. Initial coding occurred at each “meaning unit” defined as a data segment that contains one idea or theme and is comprehensible outside its context [26]. This stage of analysis was data driven and descriptive and memos were used to track the development of common codes. For T1 and T2, one researcher (ROD) coded the complete dataset and another (HFMQ) independently double coded 10% of the interviews (n = 3). This process was repeated for T3 with one researcher (HFMCQ) coding the dataset and another (ADB) independently double coding 10% of the interviews from this time point (n = 2). The codes generated by each set of coders were compared and discussed in order to challenge their perceptions, ideas and assumptions regarding the data and to develop a more comprehensive and in-depth understanding of the experiences of contact tracers [27].While there was variation in the specific labels given to codes, each set of coders agreed on the codes attributed to each piece of interview text. During the discussions, the coders re-examined the research questions and their understanding of which codes were most relevant. Following discussion, each code was given a clear, concise, and unambiguous label, a definition of its meaning and a description of inclusion and exclusion criteria [26].

Codes were then categorised and grouped into themes. This was done by grouping similar codes and exploring other connections between codes and was completed at each time point. Memos were used to track the development of these codes. Lastly, themes were defined and named. Each theme was reviewed with reference to the literature in order to aid clarity and ensure the relevance of each theme to the research questions. This was done concurrently with writing up the results of the analysis.

### Ethics

This study was conducted in accordance with the Helsinki Declaration. Ethical approval was obtained for this study from the University College Dublin Research Ethics Committee (Ref: LS-20-78). Written informed consent was obtained from all participants prior to commencing the interview. In order to de-identify interview transcripts, each transcript was assigned a unique code made up of P (participant) number, interview number (e.g., the first interview conducted within each time point was given the number 1) and the timepoint of the interview, i.e., T1, T2, T3.

## Results

Interviews lasted between 16 and 68 minutes, with an average length of 33 minutes. Participant characteristics are displayed in Table 1. Six themes were identified concerning the participants’ experiences of contract tracing. These themes were: The Contact Tracing Role; Training as Reactive, Not Proactive; Systems Issues; Workforce Culture; Motivation; Support. Related subthemes are also discussed.

**Table 1:** Participant characteristics.

Participant Information	Timepoint 1 (T1)	Timepoint 2 (T2)	Timepoint 3 (T3)
**Number of Participants**	22	8	17
**Characteristics**	Clinical background: 6 Non-clinical background: 15 Team Lead: 1*	Clinical background: 1** Non-clinical background: 6 Team Lead: 1*	Clinical background: 11 Non-clinical background: 4 Team Lead: 2*
**Time in Role**	3 months: 6 4 months: 3 5 months: 7 6 months: 3 7 months: 1 11+ Months: 2	5 Weeks: 1 5 Months: 2 6 Months: 1 7 Months: 1 8 Months: 1 9 Months: 1	14 weeks: 1 3 months: 2 7 months: 1 9 months: 1 10 months: 8 11 months: 2 12 months: 1 16 months: 1
**Age**	18–29: 6 30–39: 3 40–49: 6 50–59: 2 60+: 3 Prefer not to say: 2	18–29: 2 40–49: 2 50–59: 2 60+: 2	18–29: 5 30–39: 2 40–49: 1 50–59: 6 60+: 3

* The team lead interviewed at T1 had a non-clinical background and at T2, the team lead had a clinical background. The two team leads interviewed at time point T3 had a clinical and a non-clinical background.

** The clinical contact tracer interviewed at T2 was also interviewed at T1.

### The contact tracing role

Two sub-themes, Perceived benefits of the role and Anxiety about the role, were identified within this theme. These explored participants’ motivations and concerns related to the contact tracing role.

#### Perceived benefits of the role

Participants discussed their early experiences of working as a contact tracer. There was a sense of excitement and enthusiasm for returning to the workforce and meeting new people. Many participants spoke of the interpersonal connections they made and stated that they enjoyed the role and took pride in their work, describing it as *“very rewarding”* (P26T2). In addition to gaining specialised knowledge in relation to COVID-19, the role provided the majority of participants with valuable work experience in a new industry and conferred transferable skills. The role also benefited some participants by providing them with a new perspective and experience that built their confidence:

*“…it’s opened my eyes up to maybe something new*, *something different and it’s definitely a new thing… and I suppose it’s given me confidence as well to do something else with my life…” (P25T2)*

#### Anxiety about the role

Most participants reported some degree of nervousness on beginning contact tracing. Those who joined in the initial wave of the pandemic were particularly nervous due to the unknown aspects of the pandemic at that stage. In particular, making the first call was a source of apprehension and nervousness for most participants, and for some this had stayed with them, and came up from time to time:*“… even now sometimes when I’m about to call someone*, *I just get this wave of nervousness even though I’ve made countless calls at this point” (PT2T1)*. Those few participants who had experience of working both remotely and onsite said that working remotely was overwhelming in comparison to working onsite at a contact tracing centre (CTC) where they could quickly and more easily discuss issues and ask colleagues for advice about calls.

### Training as reactive, not proactive

There were four sub-themes within this theme; Insufficient Induction, On-the-Job-Learning, Continuous Updates from Contact Management Programme (CMP) Challenging and Suggested Improvements for Training. These explored participant’s experiences of the different aspects of training and their suggestions for improvement.

#### Insufficient induction

Before their first day in the role, contact tracers completed training via online bespoke modules. While this provided some idea of what to expect before beginning their role, and gave them an appreciation for the contact tracing computer system, many participants said that it did not fully prepare them for the role, and that they learned best when they were onsite: “*…you only start learning when you’re in the role you know” (T27T2)*. Insufficient induction training was often perceived to be due to surges in COVID-19 case numbers. As a result, more experienced contact tracers and those with clinical backgrounds mentioned supplementing training and providing ad hoc support for others as needed.

As a quality improvement approach was used to improve training through continuous adaptation based on contact tracing staff feedback (Martin et al, 2022), some participants were pleased with improvements to the induction training by T3: *“The training is good…They have very good documentation*, *very good videos I think*, *the videos I found were excellent*.*” (P5T3)*

#### On-the-Job-Learning

Online training portals and emails provided ongoing updating of information and scripts to support contact tracers. While the majority of participants said this information was well designed and useful, many felt that there was an onus on them to stay up to date with process and information changes and that, due to the nature of shift work, they could miss important information updates.

Due to surges in case numbers at T1 and T3, contact tracers with non-clinical backgrounds had experience of making calls to inform people of their positive result and felt that insufficient training had been provided to support them in this task. This caused frustration and anxiety among many non-clinical contact tracers because they were unsure of their role and the procedure for conducting calls to individuals who tested positive for COVID, which often involved talking to people who were in distress:

*“… it was too late to give us proper training. So, that was just something that was quite frustrating as well (*…*) we didn’t know what we were doing and we felt like we were calling people who were extremely stressed and upset and had all of these technical questions and we couldn’t answer them” (P20T1)*

#### Challenges of keeping up to date in a dynamic context

Some of those in the role of team lead found it difficult to balance their need to learn new work instructions to implement new public health policy, for example a change in advice such as ‘isolate for 10 days rather than 14 days’ with supporting the learning of contact tracers. Some contact tracers also felt they did not have enough time during shift hours to keep abreast of new work instructions:

*“…you’re doing a job in there and you’re seeing pop-ups coming in, new work instructions like*, *there is barely time to read these work instructions… you certainly couldn’t do it in there, I don’t feel anyway because then you couldn’t be making calls.” (P17T3)*.

The majority of participants gave positive feedback on ongoing updating of information received through group calls led by colleagues with a greater level of contact tracing experience during T1. At this time point, many participants also said they benefitted from group briefings and practical updates led by their team leader: *“…there’s a huddle every morning and they’ll announce any new changes immediately day-by-day…” (P16T1)*. At T2 and T3, as information and knowledge emerged during the dynamic situation, information updates become frequent but some contact tracers felt out of touch with developments, particularly those working part time. Those working part-time said they used these resources often as they needed to catch up on any information they may have missed, but that this was often time consuming: *“… searching for the up to date absolutely right information can be time consuming and yeah that’s the most difficult thing*.*” (CP3T3)*

#### Suggested improvements for training

There was an overall perception that the current training was “…*reactive though rather than proactive” (P21T1)*, and that a more proactive approach to training was required to better prepare contact tracers for changes to their role.

Many participants recommended that a greater focus be put on onsite learning with more one-to-one supervision and a longer period of shadowing a more experienced contract tracer. One participant suggested having a training representative to co-ordinate this for each CTC: *“… someone who is assigned to keep an eye on the training in general” (P26T2)*. *Several* participants also commented on the need for more regular and participatory information updates, particularly with regard to complex cases:

*“…they definitely could organise more little brief training sessions (*…*) how to deal with flights or how to deal with schools…” (P17T3)*

### Systems issues

There were three sub-themes in the Systems Issues theme. These were Contact Tracing System Efficiency, Feelings of Disconnection from Management Structure, and Impact of High and Low Case Numbers. These explored participants’ perceptions of the contact tracing system’s efficiency, and how it adapted to challenges over time, their feelings of disconnection from senior management, and the impact of changing case numbers on the contact tracing role.

#### Contact tracing system efficiency

The majority of participants provided positive feedback on the efficiency of the contact tracing technical system: *“…the systems that are in place are quite good…”* (P16T3), and the overall turnaround of the contact tracing process. There was also positive feedback given on the interpreter service which contact tracers use when calling individuals who do not speak English. Following on-going efforts at quality improvement, by T3, there was a general perception that the system had become more streamlined in preparation for further surges. Some participants made suggestions for improving efficiency such as automating more clear-cut call scenarios, grouping members of households/residential facilities to minimise the number of contact tracing calls made, and creating a separate call queue for unanswered calls.

#### Differences between Contact Tracing Centres (CTCs)

Many participants spoke about perceived differences between CTCs. At T1 and T2, some contact tracers were temporarily transferred from one CTC to another due to a COVID outbreak, and the HSE IT ransomware attack, respectively. At T3, contact tracers from one centre were permanently transferred to another. Comparing experiences across CTCs, Some participants appreciated CTCs that provided greater levels of clinical support, and autonomy. Some participants also noted they had a more positive experiences in CTCs where they had greater training opportunities as they generally felt like they had been facilitated to make a greater difference in people’s lives: *“…it really felt like you were making a difference*.*” (P6T3)*.

CTCs differed in terms of their culture of psychological safety. Some were perceived as work places where it was safe and easy to ask for support: *“…we have a policy of every experience is a learning experience…(P6T1)*. While others were perceived to have a culture where it was difficult to ask questions. Several participants’ perceptions that they were not listened to or invited to contribute to decisions being made, or provide feedback reflected a sense of low psychological safety:*“…if there’s something that we feel should be changed it’s like dismissed straight away (P25T2)*.

A key event which occurred within the system between T1 and T2 was the ransomware attack on the HSE IT system which occurred in May 2021. Several participants expressed frustration with the impact this had on the system, such as having to move to a new contact tracing centre, not having access to their desktop and not having emails, *“it’s terrible*, *it disrupted everything” (P26T2*). While there was initial frustration, there was a general understanding that the ransomware attack was due to an external source, and participants generally had a positive perception of the efficient recovery: *“Yes so*, *they’ve got our system back up and running and in fairness to them they did that really*, *really quickly they did get our system back*.*” (P23T2)*

#### Feelings of disconnection from management structure

While there was a generally positive perception of team leads, many participants described an organisational culture with a perceived lack of familiarity between senior managers and contact tracers: *“…the culture is coming from nameless people who suddenly make a decision” (P3T2)*.

The perceived disconnection between those making calls and those making decisions was believed to be due to multiple layers of management, and was said by many to result in a lack of trust from those in management roles, and a lack of understanding of the day-to-day nature of contact tracing.

Some participants did not feel valued and linked this feeling to the high turnover rate among contact tracers with clinical and non-clinical backgrounds. By comparison, participants appreciated it when their CTC provided greater levels of clinical support and autonomy.

#### Impact of high and low case numbers

The majority of participants highlighted the wide variation in their experience at work based on the number of cases of COVID-19. Many participants at T1 reflected on the number of calls they had to make during December 2019 and January 2020 when daily cases increased from 522 on the 19th December to 6,886 on the 10^th^ January:

*“… Christmas was busy like that was the worst I could say*, *that was the worst month anyway for me working there because we knew no one was staying in, everyone was going to their families, like no one was going to behave themselves, it was Christmas like we knew it was coming. So, it was non-stop call after call, contact after contact.” (P12T1)*

While many participants enjoyed the opportunity to be busy, there was also a perception from a few that they would have experienced burnout if the high case numbers had lasted any longer. At T3, the high numbers of calls to be made were said by several to result in a more intense and pressurised work environment:: *“…the pressure of making calls and you know*, *not leaving the desk*, *on a phone all of the time*. *That is very hard*.*” (P11T3)*. *Many* participants felt more relaxed during times of low case numbers. During times of very low case numbers however some participants found their work more tedious: *“…there’s no cases in the system*, *you’ve nothing to do*.*” (P14T3)*.

During surges in case numbers, staff with non-clinical backgrounds had to make calls to individuals who tested positive for COVID. This became a source of tension among many participants as staff without clinical backgrounds received lower pay on average than staff with clinical backgrounds. Many participants discussed their mixed feelings toward case numbers; while having high case numbers meant the virus was spreading, it also provided more job security: “…*If cases are up*, *it means I am going to be busy which means I am going to be at work for a lot longer*. *I am not going to be let go*…*” (P19T1)*. The perceived reliance of their role on the pandemic continuing and the resulting lack of job security was a source of anxiety to many.

### Workforce culture

Within the Workforce Culture theme, two sub-themes were identified: Care centre vs. Call Centre and Communication Challenges. These captured contact tracing staff’s reflections on what they appreciated in CTCs and issues in communication between contact tracing staff and senior management in the CMP.

#### Care centre vs. call centre

In the previous theme, it was evident that many participants across each contact tracing centre perceived an increased pressure from senior management to make large quantities of calls during surges in case numbers. Many participants often highlighted that they saw the service they provided as a care service but that it was often being run like a call centre, with focus on quantity of calls over quality:

*“I think there’s a bit of trend at the moment to make it just an operational system like a call centre for (telecoms company name)}*. *We would feel we are health service provider, offering health advice and that the dynamic of being empathetic and given [sic] good health advice outweighs any operational non-clinical work that needs to be done so I think that’s very important” (P6T1)*

This was a source of frustration for many and participants reported that this focus on the number of calls made did not take into account the differing types of calls, and the complexity and potentially high number of close contacts that had to be gathered. One participant who made specialised calls, which were often the most complex cases, found this pressure frustrating as they were not informed as to how to increase the quantity of their calls:

*“I’ve brought it up to them before saying I can’t do these any quicker, unless you can tell me how I can do it quicker, by all means*. *But they don’t have those answers, so I still do the same amount of work…” (P17T3)*

This focus was perceived to create a pressurised environment which many participants believed impacted the quality of calls being made: *“… when you put a workforce under that kind of pressure… there will just be*, *there will be mistakes” (P8T3*). It was also perceived to come at the expense of contact tracing staff’s wellbeing, even fostering comparisons between colleagues:

*“But it also fosters people feeling overwhelmed and anxious because they*’*re not doing as many calls as the next person…” (P7T3)*

#### Communication challenges

Many participants perceived that there was a lack of communication from senior management to contact tracers: *“I think in terms of what would be really helpful was if senior management kind of spoke to us*, *the tracers on the floor*, *and kind of put what’s happening in a national context” (P2T3)*. Many participants also felt a lack of attention to communication from senior management in terms of feedback which they found frustrating:

*“… if you have a question you have to fill out a form, that form goes to team lead, then that team lead gives that form to someone else*, *then somebody else will look at the form and then may get back to you to answer your question*. *Like, why would you bother?” (P7T3)*

These levels of communication created annoyance among many of the participants, particularly when contact tracers were not made aware of the rationale behind changes being made, which had the effect of reducing both their autonomy in their role, and the influence of team leads on informing decision making. One participant said that their team lead was *“… constantly bringing up issues because she wants calls done by clinicals but she’s kind of ignored because there’s so many layers of people making decisions” (P3T2)*.

At T1 and T3, some participants suggested that a more centralised approach to management throughout the system, with management joining the morning huddles, might improve some of the communication issues:

*“…more like, collaborative work between maybe management and the tracers you know*, *in the morning huddle or that kind of thing, where the admin staff, management staff are all involved as well…” (P12T3)*

Additionally, many participants were grateful for use of a translation service which had been made available to contact tracers for ringing members of the public whose English may not have been their first language. Many participants said that this ensured that members of the public who did not have English as a first language were able to understand the information and guidelines communicated by contact tracing staff.

### Motivation

Within the Motivation theme, two sub-themes were identified: Primary Motivations and Demotivation and Turnover. These captured contact tracing staff’s motivations and issues that they felt were demotivating and leading to increased staff turnover.

### Primary motivations

The majority of participants were motivated through seeing the role as an opportunity to return to the workplace both after losing employment due to COVID-19 restrictions, and after retirement, or as an opportunity to develop their career. Many contact tracers also referred to wanting to help the public during the pandemic as a central motivation for becoming involved in contact tracing. During surges in COVID-19 case numbers some participants were motivated to increase the number of calls they made out of a sense of duty.

#### Demotivation and turnover

The large number of calls to be made during surges in case numbers were however de-motivating for other participants: *“…it’s quite disheartening sometimes because like you might finish the day with the same amount of calls on the system as there was starting off the day and it’s just like you’ve made no progress at all” (P12T3)*.

During surges more calls were required to be made to inform people of positive results for COVID-19. Staff with non-clinical backgrounds had to begin making these calls, which had previously been made by staff with clinical backgrounds. As staff with non-clinical backgrounds received lower pay on average than those with clinical backgrounds, this became a general source of tension and demoralisation. Many participants believed that this issue was resulting in staff turnover.

At T2 and T3, the change in focus on increasing the quantity of calls was generally perceived as a culture shift and seen as detrimental to participant’s motivation, and the cause of greater staff turnover, particularly the loss of staff with clinical backgrounds: *“…they’ve become more like call centres as opposed to clinical patient call centres… and it’s why they’re haemorrhaging staff*, *clinical staff” (P3T2*). Many participants also felt the pressure to make higher numbers of calls to be demoralising. This culture shift and the resultant turnover was also linked to contact tracing staff feeling under-appreciated, inadequately compensated for their work, and lacking in formal supports:

*“…people are leaving left, right and centre because they feel unappreciated, they’re not paid properly*, *and yeah, it’s just a glorified call centre with no supports.” (P7T3)*.

### Support

There were three sub-themes under the Support theme, Peer support, Leader support and formal support mechanisms, and Support needs. These concerned the informal and formal supports available to participants and their perceived support needs.

#### Peer support

The most commonly referenced source of emotional support for participants was peers. Participants who were in an older age group (60+) and had more healthcare experience spoke of providing support to younger contact tracers, often with an aim to compensate for a perceived lack of formal psychological supports available to staff. The majority of participants spoke of supporting and being supported by their colleagues after difficult calls:

*“… if you had a difficult call you could turn around and say to a colleagues “oh that was quite difficult” and they might go you know*, *you kind of like you can have a talk about it if it was a call they had or whatever…” (P1T1)*

While peers were the most common source of support, there was a general awareness that they required more support than they could provide to each other:

*“you know, you’re bringing home all the horrible stuff of COVID because you’ve nowhere to kind of*… *apart from your colleagues. But your colleagues are tired, your colleagues are burnt out from doing the same calls as you. But we do support each other as best we can.” (P7T3)*

#### Leader support and formal support mechanisms

The majority of team leaders described encouraging their team to come to them when in need of emotional or operational support and many participants demonstrated awareness that they could go to their clinical and team leaders for practical or emotional support.

Awareness of the availability of professional, formal emotional support varied, with some participants not being aware of any formal support: *“…you can talk with your colleagues*, *but I don’t know of any supports*, *no*.*” (P9T3)*. While other participants were aware of these supports there was a general perception that their availability was poorly communicated: *“…there’s a poster there but no one’s ever actually verbally spoken about it to us…” (P1T1)*. The process of accessing helplines was perceived to be overly convoluted. Another participant was aware of the availability of a psychologist but unaware of how to make contact.

Additionally, several participants believed that the formal supports available were too basic: “*… most of us already dealt with stress in their lives*, *so we do have our own ways to deal with stress…” (P18T3*), and not tailored to aspects specific to their role, such as speaking to distressed clients on the phone:

“…*there was one or two clients who I think they were nearly suicidal and there was no training there for us in what to do in terms of somebody being that bad and then there was no support then as well for us as callers after we take the call like psychologically*…*” (P25T2)*.

#### Support needs

Participants generally spoke to a need for emotional support after difficult calls: *“I’ve had a bad call*, *an upsetting call*, *I would take a few minutes and not make a phone call*, *and talk to some colleagues” (P2T1)*. At T1, support needs differed according to age and clinical background with those in the older age category (60+), and those with a clinical background commenting on feeling like they needed less support than younger contact tracers: *“… I’ve broken worse news to people than that they’re COVID positive*.*” (P3T1)*.

At T1 there were a few examples of younger participants who had a clinical background finding certain calls particularly difficult. By T3, several participants with backgrounds in healthcare who made specialised calls which were often emotionally challenging found the lack of a formal support structure around their role particularly difficult:

*“After difficult calls, it’s just like you could write that this person was deceased and I talked to the family member and they also had COVID, that could be a note and sure nobody would even bat an eyelid*. *There are a couple there who would touch base but they don’t even know, they can’t keep up with every call.” (P17T3)*.

## Discussion

The findings of this multi-timepoint qualitative study provide a rich understanding of the experiences and needs of contact tracing staff during the COVID-19 pandemic response in Ireland. While research has explored the experiences of general healthcare staff working during the pandemic [28], there is a dearth of research on the needs and experiences of contact tracing staff during pandemics and epidemics, particularly when working in a contact tracing centre environment. Preliminary research has explored the implementation of a contact tracing system [10], and one study explored the experiences of contact tracing staff working remotely in New York [29], while another explored the psychological impact of contact tracing work on contact tracing staff during the COVID-19 pandemic [30]. As contact tracing is a central public health strategy in a pandemic response, there is a need to learn how to best support individuals in this role in the present and for future pandemic preparedness. Themes identified in this study included The Contact Tracing Role, Training Reactive-Not Proactive, Systems Issues, Workforce Culture, Motivation, and Support.

### The contact tracing role

Many participants experienced feelings of apprehension on beginning the role, particularly in the initial stage of the pandemic due to the unknown nature of the pandemic, as did many populations around the world [31]. Working in a contact tracing centre rather than remotely did however lessen participant’s feelings of stress around beginning the role. Understandably, making the first phone call was an additional source of nervousness for participants. Some participants experienced vestiges of this nervousness in the moments before making calls months later. Participants found the role provided an opportunity to develop their general abilities and skills and build their confidence in being able to take on new career opportunities.

#### Training

While there are no widely accepted evidence based guidelines for contact tracing, effective tracing requires effective health communication, cultural sensitivity and active listening [32]. Participants received induction training which was provided online and on-site, and were regularly updated on system and process changes; these processes continually evolved and adapted as part of a quality improvement approach to training [2]. Participants suggested that a greater emphasis be placed on practical on-site training with longer periods of shadowing and one to one supervision as this was perceived to better prepare them for the role. The provision of online discussion sessions and online role playing exercises [29], which can improve health communication skills and cultural competencies [33], may also help in building contact tracers’ skills and confidence in emotionally charged situations [10,34].

At times when policy and guidelines evolved at a rapid pace [35], there was an expressed need for more frequent updates of information regarding these policies and guidelines, as emphasised by contact tracing staff in previous research [10,29]. This was particularly emphasised by those working part time, however this may be due to the constraints of part-time work. Contact tracers experienced frustration and anxiety when they felt they had received insufficient training to make calls to cases. This occurred in response to a surge in case numbers. Inadequate levels of training for healthcare volunteers have been found during the early stages of past epidemics [36]. There is a need to improve the reactivity of training in order to better anticipate challenges to the contact tracing system and ensure the on-going availability of training in the evolving context and knowledge about COVID-19. Additionally, facilitating contact tracers to contribute to the development of script scenarios and other aspects of their role, and compile examples of challenging call scenarios and response strategies into reference documents [10] may have the dual benefit of increasing contact tracers sense of autonomy and confidence in their role, and improving contact tracing processes and supports.

#### Systems issues

The impact of high and low cases sub-theme highlighted how high call numbers demanded during surge periods was said to result in a more pressurised work environment. There was a perception that making the large number of calls required during the surge in COVID-19 cases over December 2020-January 2021 [19] for any longer would have led to their experiencing burnout, an outcome prevalent in healthcare staff responding to the COVID-19 pandemic [37]. Allowing for more breaks during shifts [38], and encouraging physical exercise [39] and more responsiveness to the expressed needs of contact tracers such may help to prevent the development of fatigue and burnout.

High case numbers also led to instances of role blurring when contact tracers from different backgrounds performed similar duties to staff on different salaries. Role blurring commonly occurs as a response to situational factors [40] such as during periods of increased demand on services. Improving communication around the duties of each level of contact tracer may assist in clarifying the boundaries of contact tracing, and their differing levels of pay, in addition to providing opportunities for career development for contact tracing staff without clinical backgrounds.

While high case numbers were seen as a negative, they were paradoxically also seen as an indication that the role of contact tracing would continue. This seeming reliance on the pandemic continuing was a source of uncertainty and anxiety for participants. Interestingly contact tracers in a US-based study found the prospect of a guaranteed year of employment a source of motivation for applying to be a contact tracer, due to high levels of COVID-19 related unemployment [29]. Similar issues around the sustainability of the contact tracing role were raised by volunteer contact tracers in previous research who did not see a volunteer-driven program as sustainable during the “maintenance phase” following the crisis [10]; high staff turnover rates were noted in this previous research as volunteers took up paid employment opportunities. The provision of staff development and outlining of career pathways in public health for contact tracing staff may be useful in retaining contact tracing staff. The development of a long term national contact tracing strategy may also help in reducing staff turnover by providing more stable employment prospects and develop a skilled service to reduce and contain the current pandemic and future scenarios [32].

#### Communication challenges and workforce culture

Within this theme, a key sub-theme was the contrast drawn between experiences of contact tracing as a care centre versus a call centre. Contact tracers perceived their role as providing a care service. However, many contact tracers perceived that contact tracing management appeared to view their role more akin to that of a traditional call centre staff, with a focus on the quantity of calls they made. This focus on quantity however was a source of frustration to participants who felt a need to show empathy and build trust and rapport with people facing a new and potentially frightening illness, as did COVID-19 contact tracing staff in previous research [10]. Participants felt that the emphasis on quantity did not take into account this need to support members of the public nor the differing call types and the complexity of calls they often had to make. A similar tension between quantity and quality was found between nursing staff who worked in a health service related call centre and their management, with nurses outlining their need to provide a patient oriented service rather than a commercial service [41]. The focus on quantity was perceived to create a pressurised environment, which participants believed impacted the quality of calls they made, and the wellbeing of contact tracing staff. This focus was said to be demotivating and an issue of frustration, with participants believing it was linked to the high rate of turnover of clinical staff.

From a public health point of view, there is an essential need to inform cases and contact trace in a timely manner as reducing the delay in testing for COVID-19 has been found to have the one of the greatest impacts on reducing further transmission of the virus [42]. This created a discrepancy between what was required for the role in terms of public health, particularly during times of high call numbers, and what was required by staff to support their wellbeing. Communicating the need to inform cases and contact tracers in a timely manner may go some way toward easing some of this tension, however there may also be a need to balance the requirements of the contact tracing system and those of contact tracing staff in order to support their wellbeing and reduce turnover.

The participants’ perception of poor communication between management at the national level and staff at CTCs, in addition to the perception that there were multiple layers of management in the contact racing system was another source of dissatisfaction among staff, the combination of which was perceived to result in staff frustration, less autonomy, and less ability to provide feedback. Additionally, some participants mentioned that the work culture resulted in their not feeling valued by management at the national level, a feeling to which they also attributed increased staff turnover. Professional recognition, whether tangible or intangible, is an important factor in healthcare workers’ motivation during the pandemic response [43]. As inferred by the participants, a move toward a flatter organisational structure [44] may aid in improving motivation, autonomy and the reduction of staff turnover.

The communication issues discussed by participants echoed those experienced by frontline healthcare staff during the COVID-19 pandemic with staff desiring more two-way communication and opportunities to provide feedback and contribute to decision making [45]. Previous research indicates the importance of contact tracers having direct communications with supervisors, and receiving quick responses to their questions through a chat application [10]. The promotion of two way communication may also improve the relationship between management and contact tracing staff [44]. The provision of internal communication structures could be used to promote information exchange, and shared learning between contact tracers and, as mentioned by participants, to better identify trends [10].

Many of the experiences and challenges differed between CTCs. Participants valued experiences in CTCs that provided good levels of clinical support, autonomy and more training opportunities, factors which contributed to participant’s pride and facilitated them in contributing to the pandemic response. A greater focus on increasing call volumes in CTCs presented a culture shift that was perceived to result in greater staff turnover. A way to improve on these challenges and the workforce culture throughout the contact tracing system, could be to use the processes and training opportunities in CTCs favoured by participants as a model to emulate throughout the system.

#### Motivation

In line with the findings of Shelby et al. [10] and Santella et al. [29], participants were primarily motivated to begin contact tracing by a desire to contribute to the COVID-19 response and by a desire to return to the workplace or as an opportunity to change career.

During times of high case numbers, the motivation to contribute to the COVID-19 response was an additional driver for some participants spurring them on to increase the number of calls they could make per day. Conversely, the high number of calls required during these times was de-motivating for other participants as they perceived their efforts to have little impact on the overall number of calls to be made.

A significant source of demotivation for staff without clinical backgrounds was when they needed to make calls to inform people of positive results for COVID-19, which had previously been made by staff with clinical backgrounds. The differences in pay levels between staff with and without clinical backgrounds was also perceived to cause tension. This role blurring, in addition to the increasing numbers of calls to be made as case numbers rose were believed to result in increased staff turnover among staff with and without clinical backgrounds as staff felt under-appreciated, and inadequately compensated for their work. A number of methods could be used to improve staff motivation and decrease turnover, including highlighting the appreciation of the public for their work, supporting opportunities for career advancement, and increasing managerial support [46].

#### Support

The feelings of reward participants felt from contributing to the pandemic response and the gratitude they experienced from members of the public on calls may act as protective factor against burnout symptoms [47] the presence of which has been found in varying degrees in contact tracing staff in Ireland [30]. Gratitude from members of the public has increased worker’s sense of fulfilment during past epidemics [48], however, deriving meaning from work may not be sufficient to protect against the development of adverse mental health outcomes [36].

Participants most commonly referenced their peers as a source of emotional support. During the COVID-19 pandemic and previous epidemics, peers were a key source of technical, educational, and emotional support for varied healthcare workers, with buddy systems often employed and opportunities provided for informal group reflection [49]. Previous research with COVID-19 contact tracers also highlighted a need for a buddy system, and regular peer meetings to share experiences from calls [10].

Participants appreciated peer support as it could be provided directly after difficult calls when participants felt a need to speak with someone about their immediate experience, as mentioned by healthcare workers during the COVID-19 pandemic [45]. Social support has a positive impact on psychological health [50,51] and is considered an important resource in alleviating mental distress for nurses [52,53]. It can improve psychological health by helping individuals to reduce perceived severity of the problem and the adverse effects of stress [54]. There was however a recognition that participants needed more support than they could provide to each other as they were experiencing similar levels of fatigue and burnout.

Participants’ awareness of the availability of formal emotional supports varied, to the degree that more experienced contact tracing staff provided support to their peers to compensate for a perceived lack of formal supports. For those participants aware of the availability of these supports, there was a general sense that they were poorly communicated and overly complicated to access. This echoes findings in varied healthcare staff during the COVID-19 pandemic who were often unaware of the availability of professional psychological support due to unclear and inconsistent communication of available supports [45].

For those participants aware of these formal supports, there was a perception that they were not tailored to needs specific to the role such as how to speak to distressed clients on the phone and what to do after such a call. While participants generally felt a need for emotional support after difficult calls, those with healthcare backgrounds and those in the older age category felt they required less support at T1. By T3 participants who made specialised calls felt a greater need for support after difficult calls. Ali et al [55] acknowledged the need for psychological supports for healthcare workers in Ireland and the inadequacy of counselling services provided for healthcare workers during the pandemic. They highlighted the need for enhanced availability of services for those that may require additional support and underscore the potential role that online psychological interventions [56] may play in providing this support.

While mutual peer support is beneficial and was appreciated by participants, this is best used as part of a larger support structure in order to avoid placing adverse pressures on peers when they are also experiencing feelings of burnout [45]. Team leads mentioned encouraging their teams to approach them for emotional support and participants spoke of their supervisors as being accessible for this. The provision of group reflections led by team leads such as Schwartz Rounds may help contact tracing staff to reflect and discuss the emotional impact of their work [57]. As avoidance can be a symptom of trauma, there is a need for team leaders to be proactive in checking in on contact tracing staff in order to attend both these reflections [58] and the psychological first aid sessions provided for contact tracing staff [59]. Additionally, the availability of existing supports, such as Employee Assistance Programme and the programmes that support stress management skills should be better communicated to current contact tracing staff, and during the on-boarding of new staff. Table 2 presents a summary of the key recommendations arising from this research, based on the experiences and needs of contact tracing staff.

**Table 2:** Recommendations based on learning from participants.

Theme	Recommendation
Role	Provide continual skill development opportunities.
Training	Longer period of in-person, practical training, supplemented by online supports. Dedicated time for on-going updating of information for staff during dynamic and rapidly evolving situations.
Overall systems issues	Increased receptivity and responsiveness to hearing feedback from staff in order to become more flexible to staff needs to improve system efficiency
Communication Challenges and Workforce Culture	Greater emphasis on the call types and levels of complexity of calls made by contact tracing staff, and to improve communication from management to contact tracing staff regarding the duties of the role. Huddles/regular briefings that encourage input from those making the calls to ensure processes are responsive to public needs. Openness to learning about what works well from those making the calls and to adopt processes valued by contact tracing staff in some contact tracing centres (e.g., greater levels of clinical support, and levels of autonomy) as a model to emulate across the wider contact tracing system.
Motivation	Feedback public’s appreciation of contact tracing staff. Provide opportunities for career advancement, and increase managerial support.
Support	Enhanced and more frequent communication regarding the availability of formal psychological support services and improved informal (peer) support systems for staff.

### Strengths and limitations

A strength of this research was the broad overview of contact tracing staff’s experiences recorded in real time over a six month period that included motivations and training, systems issues, communication challenges and support. The time line of the study may also present a limitation; the pandemic situation continues at the time of writing this article, and so these findings may not represent the overall experiences of contact tracing staff during the COVID-19 pandemic. However, these findings will provide greater understanding of the experiences and impact of working in the contact tracing role during COVID-19 pandemic response. A further strength of this research was in the organisation of its themes, which facilitates the implementation of the study’s findings into practice. At each of the three time points rapid reviews of the study findings and recommendations were disseminated to the CMP in the form of evidence briefs to aid the development of appropriate supports for contact tracing staff.

## Conclusion

Overall, participants were highly motivated to contribute to the pandemic response, found the role rewarding and believe they attained valuable transferable skills through this work experience. They found the training to have improved over time but desired more onsite induction training, more regular updates to changes in the system, and more proactive training. The contact tracing system was perceived to be efficient, but the precarity of the role was an issue for participants, as was the management structure and communication within the system. Working culture and communication issues discussed related to participants’ frustration with a lack of timely communication, lack of opportunity for feedback and involvement in process changes, feelings of low autonomy, and a perception of high staff turnover. There was a need for clearer communication of the availability of formal emotional supports, and the provision of informal group reflection. While participants were motivated to contribute to the response to COVID-19, role blurring resulted in some feeling demotivated in addition to the need to make increasing numbers of calls during surges in case numbers in order to reduce the spread of COVID-19. The provision of career advancement opportunities and increased managerial support may go some way to improving motivation. These findings have important implications for contact tracing practice and this research articulates a set of key recommendations (see Table 2) to support on-going system and process improvements and suggestions on how best to support the emotional and practical needs of contact tracing staff during the COVID-19 pandemic response. These findings may assist in the development of contact tracing policies, and in the development of national contact tracing systems. Additionally this study will inform researchers on the experiences of contact tracing staff during a pandemic response. Future studies should focus on how contact tracing staff motivation might be improved during times of high case numbers.
